# On Some Exotics Which Endure the Open Air in Devonshire

**Published:** 1811-03

**Authors:** A. Hawkins


### *On some Exotics which endure the open Air in Devonshire. By* A. Hawkins, *Esq.*

(From the Transactions of the Horticultural Society of London.)

In October, 1795, a Camellia Japonica was planted, (in the South Hams of Devonshire) among other shrubs, in the open ground; it has stood every winter since, without the smallest shelter, thrives well, and has never had a branch

---

or or leaf injured by the weather; it is now about four feet high, the size of a gooseberry bush, but has not flowered.

Two plants of the Fuchsia Coccinea were planted about four years ago under a brick wall, facing the South. At first the branches suffered by the frost, but they put forth new shoots in the spring, with much strength, and have flowered well every summer. During the two last years I was absent, but I understand that only the extremities of the branches were injured, and they have always flowered in great perfection.

Some plants of the Solanum Pseudo Capsicum, or Amomum Plinii, are also under a brick wall, (but not nailed against it), which have stood many years, and only a small part of the extremities of their branches has been injured by frost.

Myrtles of every kind (even the double-blossomed and orange) do exceedingly well in the open ground, though the *Silver*, from the richness of the soil, soon becomes plain.

The Buddleia Globosa likewise stands the climate, and some of the plants are ten feet high, spread wide, and make a handsome appearance. One of them is placed in a situation open to the North-east winds, where the sun cannot shine during the short days, yet it has stood there since 1794, and never had more than the extremities of the branches hurt.

About two miles from my house is the small sea-port town of Salcombe, just between those two well-known points, the Prawl and Bolt-head; the latter of which is in the parish whence this letter is written, a place that the sea washes on three sides. Perhaps of all spots in the British isles, Salcombe is the very first for climate and shelter. The celebrated Doctor Huxham used to call it the Montpellier of England. In 1774, a large American Aloe, only twenty-eight years old, and which had always stood in the open ground, without covering, flowered there; it grew to the height of twenty-eight feet, the leaves were six inches thick, and nine feet in length, and the flowers, on forty-two branches, innumerable.

Several plants of the Verbena Triphylla are growing at Salcombe in the open ground, and are now six feet high. I have not tried any of them myself; but as I expect to be more at home in future, than for some years past, I shall not fail to add this plant to those tender shrubs already growing around me.

Oranges and Lemons, trained as peach trees against walls, and sheltered only with mats of straw during the winter, have been seen in a few gardens of the South of Devonshire for these hundred years. The fruit is as large and fine as any from Portugal. Some lemons, from a garden near this place, were about thirty-five or forty years ago presented to the King by the late Earl Poulett, from his sister Lady Bridget Bastard, of Gerston; and there are trees still in the neighbourhood, the planting of which I believe is beyond memory. The late Mr. Pollexfen Bastard, uncle of the M. P. for Devon,) who had the greatest number of oranges and lemons of any one in this country, remarked, above thirty years since, that he found trees raised from seed, and inoculated in his own garden, bore the cold better than oranges and lemons imported.

